# Oligomerization and Conformational Change Turn Monomeric β-Amyloid and Tau Proteins Toxic: Their Role in Alzheimer’s Pathogenesis

**DOI:** 10.3390/molecules25071659

**Published:** 2020-04-03

**Authors:** Botond Penke, Mária Szűcs, Ferenc Bogár

**Affiliations:** 1 Department of Medical Chemistry, University of Szeged, H-6720 Szeged, Hungary; szucs.maria.1@med.u-szeged.hu (M.S.); bogar@sol.cc.u-szeged.hu (F.B.); 2 MTA-SZTE Biomimetic Systems Research Group, University of Szeged, H-6720 Szeged, Hungary

**Keywords:** Alzheimer’s disease, amyloid β oligomers, tau oligomers, physiological actions, pathophysiology, amyloid formation, AβO-TauO crosstalk, amyloid structure

## Abstract

The structural polymorphism and the physiological and pathophysiological roles of two important proteins, β-amyloid (Aβ) and tau, that play a key role in Alzheimer’s disease (AD) are reviewed. Recent results demonstrate that monomeric Aβ has important physiological functions. Toxic oligomeric Aβ assemblies (AβOs) may play a decisive role in AD pathogenesis. The polymorph fibrillar Aβ (fAβ) form has a very ordered cross-β structure and is assumed to be non-toxic. Tau monomers also have several important physiological actions; however, their oligomerization leads to toxic oligomers (TauOs). Further polymerization results in probably non-toxic fibrillar structures, among others neurofibrillary tangles (NFTs). Their structure was determined by cryo-electron microscopy at atomic level. Both AβOs and TauOs may initiate neurodegenerative processes, and their interactions and crosstalk determine the pathophysiological changes in AD. TauOs (perhaps also AβO) have prionoid character, and they may be responsible for cell-to-cell spreading of the disease. Both extra- and intracellular AβOs and TauOs (and not the previously hypothesized amyloid plaques and NFTs) may represent the novel targets of AD drug research.

## 1. Introduction

Alzheimer’s disease (AD) is an incurable and progressive neurodegenerative illness. It is characterized with memory loss, behavioral dysfunction, and mostly rapid cognitive decline. AD begins after age 65 (late-onset AD, LOAD) in most cases (90–95%). In rare cases, AD occurs at a relatively young age (< 65) constituting early-onset AD (EOAD).

Despite more than 30 years of intensive research, the pathophysiology of AD is not completely understood yet. This fact might be the reason why a very large number of complex clinical trials have failed and there is no curative treatment of the disease.

The pathological hallmarks of AD are well-known, the disease has a specific neuro-pathological profile: accumulation of proteinaceous deposits in the brain—amyloid plaques (containing β-amyloid (Aβ) peptides) and neurofibrillary tangles (NFTs). More than 80% of AD patients also show cerebral amyloid angiopathy (CAA) pathology, in which brain Aβ deposits have been demonstrated in the wall of small-to-medium blood vessels and meninges. AD is also characterized by synaptic dysfunction, neuronal loss, vascular alterations, and astrogliosis [1].

Many hypotheses have been published for explaining the pathology and progress of AD during the last 30 years. We address only the most important and accepted hypotheses:

1. The acetylcholine hypothesis: loss of cholinergic neurons and neurotransmission causes AD [2].
2. The β-amyloid cascade hypothesis [3] evolved and changed much over the years: amyloid plaques containing misfolded Aβ play a decisive role in AD pathogenesis. Aβ also drives tau-mediated neurodegeneration. The Aβ oligomer hypothesis represents the modern version of this theory [4].
3. The tau hypothesis [5]: abnormal phosphorylation of tau proteins is in the background of AD progress.
4. The dual cascade hypothesis: cellular processes simultaneously drive tau and Aβ pathology in the cortex [6].
5. The mitochondrial dysfunction hypothesis [7].
6. The neuroinflammation hypothesis [8] emphasizes the role of immune attack in neuronal loss.
7. The vascular dysfunction hypothesis [9] underlines the role of brain circulation and endothelial-mediated processes in AD pathogenesis.
8. Peripheral Aβ hypothesis: β-amyloid peptides enter the brain from the blood from the periphery [10].
9. The cellular hypothesis [1] represents a complex view of AD pathogenesis, which emphasizes the central role of aging as the major driver of the disease and the participation of different brain cells (microglia, astrocytes, and brain vasculature) in the pathophysiology.

The basic pathophysiology and neuropathology of AD have been widely reviewed [11,12,13,14,15,16]. Amyloid forming proteins play a crucial role in AD pathogenesis [17]. It has been accepted that brain accumulation of Aβ peptide and hyperphosphorylated tau protein aggregates may initiate the pathological processes. It was considered that the imbalance between Aβ synthesis and clearance, and thus Aβ accumulation, initiate AD. Homeostasis disturbances in aging might be the most important factors in AD dementia. As amyloid plaque precedes cortical tau pathology, it was considered that Aβ drives tau-mediated neurodegeneration in AD. There is strong evidence that Aβ and tau aggregation results in toxic assemblies (soluble oligomers), and effective inhibition of the early steps of aggregation might prevent disease progression [18]. It is now generally accepted that these toxic soluble Aβ and tau oligomers play a key role in AD pathogenesis [17,19,20,21]. Oligomeric Aβ (AβOs) and tau (TaOs) proteins may develop into intra- and extracellular assemblies, both forms can trigger AD pathogenesis. It has also been accepted that Aβ oligomers, but not monomers or fibrils, are responsible for the pathophysiological events of AD [22]. It has been demonstrated very recently that the levels of gene activity changed dramatically as Aβ and tau accumulated in the brain [23].

Over the last 25 years, many small molecular drugs and neutralizing antibodies have been designed and prepared that decrease the formation of Aβ peptides or bind Aβ and tau fibrils. Some drugs were also clinically tried in the treatment of AD. Until now, all the clinical trials have failed, and interestingly, some of the drugs worsened the cognitive status of AD patients [24]. The failure of Aβ- and tau-targeting therapies and the presence of amyloid plaques in cognitively healthy persons question the original amyloid cascade hypothesis.

In this review, we summarize the most important results of AD research, focusing on the physiological role, structure, and toxic effects of different Aβ peptide and tau protein assemblies.

## 2. Amyloid Peptides

### 2.1. The Physiological Role of β-Amyloid Precursor Protein (APP) and the Monomeric Aβ Peptide

Aβ peptides represent short (37–43 amino acid (AA)) segments of a transmembrane glycoprotein, called the β-amyloid precursor protein (APP). Recently, several reviews have been published on the structure and the complex physiological functions of APP [25,26,27,28].

The processing of APP by proteolytic enzymes (α-, β-, and γ-secretases) results in a complex mixture of soluble protein fragments and peptides reviewed in [25]. The main proteolytic cleavage starting by α-secretase provides ”soluble APPα” (sAPPα) and an 83 AA peptide fragment (non-amyloidogenic pathway). The minor, amyloidogenic pathway (the cleavage of APP first with β- then with γ-secretase) results in the formation of “soluble APPβ” and β-amyloid peptides of different chain length (37–43 AA). Very recently, cryo-electron microscopy (EM) studies have revealed the structure of γ-secretase at atomic resolution [29]. This model can be used for explaining the fine details of Aβ formation from APP.

Amyloidogenic cleavage of APP occurs in membranes, where APP, β-, and γ-secretases locate together, most probably in the cell membrane and intracellularly in the lysosomes [30]. γ-secretase is a complex aspartyl protease. Its catalytic subunit (presenilin-1 (PSEN-1) or presenilin-2 (PSEN-2)) contains two aspartyl residues. Mutations in APP as well as PSEN-1 and PSEN-2 genes modulate amyloidogenic cleavage and may influence the production and toxicity of Aβ peptides (see Section 4).

The physiological functions of APP also include cell adhesion, synaptogenetic activity, and control on brain development. APP shows positive neurotrophic effect, plays a crucial role in Wnt signaling, and can function as a receptor [31].

Full length (Aβ 1-40, Aβ 1-42) peptides were considered earlier the most abundant forms in the complex mixture of Aβ peptides. Very recently, high-resolution mass spectrometry has demonstrated that truncated, shortened peptides are the main Aβ components in the brain [32]. Altogether, 26 Aβ proteoforms have been identified from AD brain and cerebrospinal fluid (CSF) [33]. One of the N-terminal truncated forms is the pyroglutamylated peptide, a very toxic Aβ proteoform [34] that may drive Aβ misfolding and aggregation.

Monomeric Aβ should have important biological functions in low (physiological) concentrations. Evolutionally, the Aβ is a very old (around 500 million years), conserved molecular sequence. All vertebrates have APP and β-secretase; and the sequence homology between human and other mammalian Aβ is very high (= 95%) [35]. The survival and activity of Aβ during the long evolution process of animals proves that Aβ provides a special advantage for survival of individuals. It was found that depletion of Aβ had bad consequences in animal models and also in humans [36]. Aβ knockout mice survived, but their brain development and neurogenesis were disturbed [37].

Monomeric Aβ peptides may play beneficial roles in human physiology [36] having multiple physiological actions:

1. Regulation of synaptic function by activating nicotinic ACh receptors and participation in memory consolidation. Aβ monomer homeostasis is essential for normal synaptic function [38].
2. Promotion of recovery after traumatic brain injury.
3. Protection of the blood-brain barrier by blocking leaks and preventing leakage.
4. Antimicrobial activity, probably by disturbing membrane structures of microbes.
5. Suppression of tumor growth through inhibition of viruses.

The concentration dependence of the physiological effects of Aβ peptide shows controversial effects: it helps memory consolidation at low (picomolar, physiological) concentrations; however, it inhibits memory at high (pathological) concentrations [39]. As a consequence, Aβ in physiological concentration improves memory. Over a critical concentration (nanomolar range), the monomers start to aggregate to different AβOs [25]. Several negative clinical experiments with drug candidates (e.g., the failure of β- and γ-secretase inhibitors that decrease Aβ levels) could be explained with the missing neuroprotective effect of monomeric Aβ.

### 2.2. Formation of AβOs and Fibrils by Aggregation

**Figure 1:**
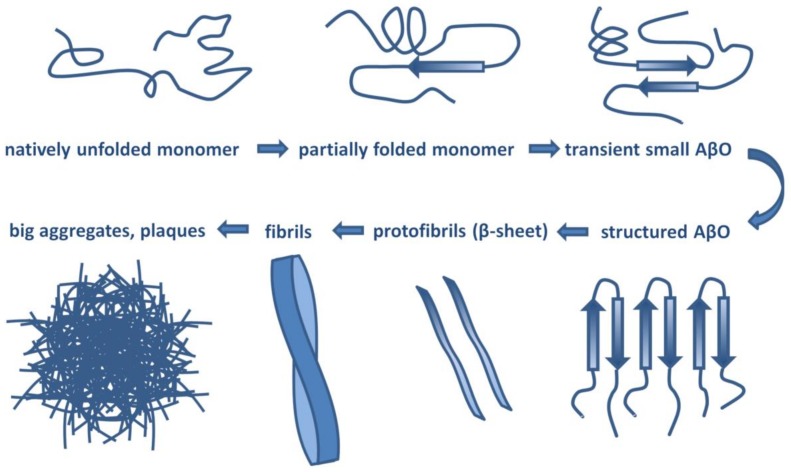
Structural assemblies of Aβ peptides. AβO: Aβ oligomer.

Aβ has a molecular lifecycle from monomeric via oligomeric forms to fibrils. Aβ is an intrinsically disordered protein (IDP) lacking a stable 3D structure. IDPs can associate with other molecules and form different fixed structures. The structural “evolution” of Aβ peptides may result in assemblies with growing size shown in Figure 1.

The amyloid oligomer hypothesis was first introduced in 1998 and was based on the discovery that fibril-free preparations of AβO were neurotoxic and could cause neuronal cell death [4].

Since this time, over 4000 scientific papers have been published on AβO, among them more than 400 reviews. Recent reviews by Selkoe and Hardy [19] and Cline et al. [20] give an excellent summary on the development of the AβO hypothesis.

There is no universal definition for AβOs; in general, these heterogeneous and transient assemblies contain sub-100-mer aggregates [40].

Many laboratories all over the world have tried to isolate well-defined AβOs with fixed structure and reproducible pathogenicity. However, it is an almost impossible task owing to the transient nature of AβOs. Different laboratories gave different names to the oligomeric assemblies of Aβ 1-40 and Aβ 1-42 (reviewed by Chiti and Dobson [18]):


⋅dimers and trimers

⋅protofibrils (PFs)

⋅pentamers

⋅annular protofibrils (APFs)

⋅dodecamer (Aβ + 56)

⋅amyloid derived diffusible ligand (ADDLs)

⋅globulomers

⋅prefibrillar oligomers (PFOs)

⋅amylospheroids (ASPDs)

⋅spheric amyloid intermediates


The term of Aβ oligomer can be defined as an assembly of misfolded peptides that maintain the solubility. AβOs are polymorphic in size: some of them may have very high molecular weight (up to 500 kDa), and all of them are soluble. None of them have a specific shape, and they never possess cross-β structure. Each AβO structure represents one of the early intermediates of the amyloid formation pathway [41].

AβO assemblies can be classified into two groups: toxic and non-toxic subpopulations. There are big differences between the two groups (see Table 1) [42].

The highly toxic oligomers represent only a minority [43] of the heterogeneous AβO assembly in the human brain [19]. Only an ultrasensitive assay (BioBarcode) can detect the attomolar level of AβOs in the CSF of AD patients, where the median level is 30-fold higher than that of healthy humans [44].

Several intrinsic and extrinsic factors may trigger Aβ oligomerization. The N-terminal amino acid sequence has a big influence on self-aggregation of human Aβ into oligomers [45].

Aβ of rodents does not show high propensity for aggregation and formation of β-folded structures, owing to the differences in three amino acids in the sequence: R5G, Y10F and H13R. Aggregation rate of Aβ is controlled by the β-content of the monomeric state [46].

Peptide-lipid interactions (e.g., with the ganglioside GM1 or with membrane-lipid rafts) and high Aβ concentration may start Aβ oligomer formation. ER-stress may also cause Aβ aggregation [47]. The hydrophobic surface of amyloid fibrils also catalyzes the formation of highly toxic, β-sheet-rich AβOs [48]. It has been demonstrated very recently that aggregatin, a brain protein, may interact with Aβ and facilitates Aβ aggregation [49].

Fibril formation begins with formation of different metastable oligomers [50,51]. Mature Aβ fibrils represent the final stage of fibrillogenesis. More and more evidence demonstrates that truncated Aβ peptides drive AβO and fibril formation [34]. The complex process of fibril formation involves primary and secondary nucleation, fibril elongation, and fragmentation. Primary nucleation is a process which begins with the formation of small aggregates (metastable oligomers) from monomers and is continued with growth of fibrils by monomer addition [50,51,52]. Secondary nucleation is a reaction in which the formation of nuclei is catalyzed by existing seeds (pre-formed aggregates) composed of the same monomers. The effect of in vivo conditions on Aβ aggregation (crowded environment, pH changes, oxidative stress, presence of carbohydrates, lipids, chaperone proteins, etc.) was excellently reviewed [53].

### 2.3. The Structure of Aβ Species

#### 2.3.1. Structure of Aβ Monomers and Oligomers

Aβ monomers have no stable 3D structure, they retain conformational freedom. Monomeric Aβ 1-40 and 1-42 peptides in solution possess very similar Ramachandran-map distribution, which demonstrates a random-coil-like structure [54]. Monomeric Aβ 1-42 may have partly disordered, partly helical structure in apolar solvents [55]. However, it has mostly a β-sheet structure in neutral aqueous solution [56].

Oligomer formation is largely a hydrophobic collapse. Although the exact structure of the highly bioactive AβO is not known yet, theoretical considerations support a β-sheet-rich conformation with a high number of hydrophobic residues on the oligomer surface ready for membrane interactions. AβOs are intermediates between the monomers and fibrils.

Association of the molecules to oligomers results in decreased conformational freedom. Owing to the transient nature of AβOs, many polymorphs exist, and their structure and aggregation grade depend on the experimental conditions. As the number of molecules in the AβOs stabilizes the β-sheet structure, the average content of β-sheets increases nearly in parallel with the size of the AβO [18]. The size of AβOs that start primary and secondary nucleation in Aβ-fibril formation was determined [57]: an Aβ trimer was proved to be the primary and an Aβ dimer the secondary nucleus. In vitro experiments demonstrated that Aβ 1-42 forms pentameric and hexameric disks at 4 °C with very low β-sheet content [58]. Soluble globular oligomers were formed with mixed parallel and antiparallel β sheet structure in the presence of aliphatic detergents [59]. Solid-state and solution NMR studies of AβO structure demonstrated that high molecular weight AβOs show β-sheet conformation in the N-terminus of the peptide [60].

#### 2.3.2. Structure of Fibrillar Aβ (fAβ)

The structural transition of pathological AβO to fibrils has been studied very recently [61] and has been widely reviewed [62]. Aβ fibrils are structurally ordered non-crystalline, water-insoluble substances. Fibrils can be several micrometer long with a width of 10–20 nanometer and are visible in transmission electron microscopy [63]. The fibrils are often twisted.

Fibrils show polymorphism at the molecular level: the same protein may form fibrils of different molecular structure and morphology [18]. Different fibrils may show different toxicity, seeding, and spreading [27,64]. Different Aβ structures were found in rapidly progressive and slowly progressive AD brains [65].

The determination of fAβ structure has been a challenge owing to its structural polymorphism. Several physicochemical, spectroscopic, and biochemical techniques (solid-state NMR, high-speed atomic force microscopy [66], transition microscopy, Raman and 2D infrared spectroscopy [67], X-ray fiber diffraction, small angle neutron scattering [68,69], cryo-EM) [70] have been used for structural analysis of AβOs and fibrils. Computational studies have also been used for studying conformational dynamics and stability of different Aβ assemblies [71,72]. These studies demonstrated that fAβs have a cross-β structure [73]. Solid-state NMR studies of Aβ peptides showed that dimeric subunits construct the fibrils. Aβ 1-40 monomers have a U-shaped structure stabilized with a salt bridge within the fibrils [74,75]. In Aβ 1-42 fibrils, the monomers possess either an S-shaped [76] or U-shaped [77] structure; the latter is less stable and frequent.

**Figure 2:**
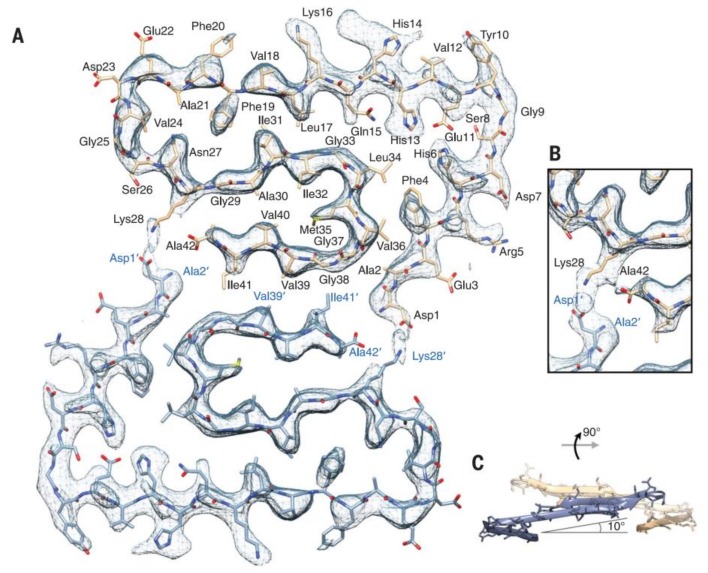
Atomic model and superimposed electron microscopy (EM) density of the Aβ fibril cross- section. (**A**) Two subunits, one from each protofilament, are shown (blue and brown) together with the masked EM density map (at contour level of 1.5 σ). (**B**) Detailed view of the interactions between the N- and C-terminus and the sidechain of Lys28 (at contour level of 1 σ). (**C**) Side view of the same two opposing subunits showing the relative orientation of the non-planar subunits. The large peripheral cross-β sheet is tilted by 10º with respect to the plane perpendicular to the fibril axis. From [70]. Reprinted with permission from AAAS.

Recently, the subatomic structure of Aβ 1-42 fibrils has been determined by cryo-EM to 4.0 Å resolution (Figure 2) [70]. The fibrils are composed of two intertwined protofilaments, and this structure agrees with the previously reported Aβ structure. Very recently, pure β-amyloid fibrils have been isolated directly from human AD brain tissue samples, and their structure was studied with cryo-EM [78]. Mass spectrometric analysis of the fibrils showed that the main components had different chain lengths (Aβ 1-40, Aβ 1-38, Aβ 2-40, Aβ 1-37, Aβ 1-36, Aβ 1-39), and the Aβ 1-42 content was low. These brain-derived Aβ fibrils showed multiple fibril morphologies (polymorphy), and all of them had a right-hand twisted structure. In contrast, in vitro formation of Aβ 1-40 and Aβ 1-42 fibrils were left-hand twisted. These results underline the priority of the use of brain-derived Aβ samples in structural studies.

### 2.4. Mechanisms of Toxicity Induced by AβOs

In the early experiments, Li and Selkoe demonstrated that soluble Aβ fraction extracted from human brain reduced long-term potentiation (LTP) and increased long-term depression (LTD) [79].

Recent data have shown that rather AβOs than amyloid plaques may play a key role in AD pathogenesis (reviewed in [21]).

**Figure 3:**
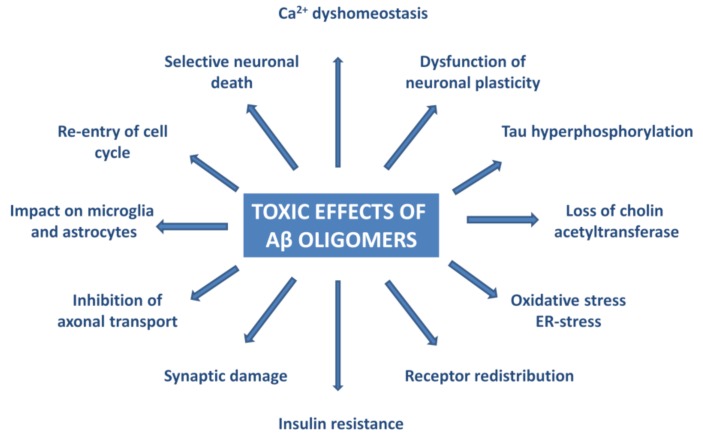
Pathophysiological effects of Aβ oligomers.

Experiments have proven that AβOs trigger tau pathology [80], cause oxidative and ER-stress and neuroinflammation [81,82,83,84,85,86,87,88,89,90,91], deterioration of synapses, and neuronal death [92,93]. Figure 3 summarizes the effects of AβO in AD neuropathology.

AβOs were observed in the synapses of AD model mice by high resolution imaging techniques (STORM, immunogold-TEM, FRET) [94]. In humans, the Osaka mutation of APP (E693 deletion) causes accumulation of AβO and AD pathology, without senile plaque formation [85,89,95,96,97]. AβO-selective antibodies prevent AD-like pathology in mice [98,99,100,101,102,103]. This evidence demonstrates that AβO is necessary (and also sufficient) for triggering AD-associated neurodegeneration.

Different hypotheses may explain the toxic effect of AβO to neurons:

1. The membrane hypothesis focuses on the lipid membrane–AβO interactions. AβOs can deteriorate membrane structures, insert directly into cell membranes, and may form pores [104] inducing Ca-influx to the cells [104]. The ganglioside GM1 plays a decisive role in the membrane–AβO interactions and AβO toxicity [105].
2. According to the receptor hypothesis, AβOs are pathogenic ligands that can bind to specific receptors [106,107,108,109]. Several facts support this hypothesis:
  - saturation of the binding sites,
  - binding specificity for definite neural cells,
  - targeting of synapses,
  - receptor antagonists inhibit the toxic effect,
  - the binding sites are trypsin sensitive proteins

Thathiah and De Strooper published that 22 G-protein coupled receptors are involved in the pathogenesis of AD [110]. Godoy in 2014 showed five anti-aging signaling pathways (Wnt, 5′AMPK, mTOR, Sirt1, and PGC-1alpha) that have a crosstalk in AD [111]. It is widely accepted that AβOs interact with several putative receptors at the excitatory synapses and thereby stimulate intracellular signaling pathways resulting in LTP inhibition and facilitation and finally synapse loss.

Up to now, a huge number of candidate AβO receptors and binding proteins have been published (reviewed in [20]). Some of them are considered to directly bind AβO:

- Cellular prion protein (PrPc)
- Na,K-ATPase alpha 3 subunit (NKAa3)
- RAGE receptor
- Tyrosine kinase ephrin type A receptor
- Tyrosine kinase ephrin type B receptor
- p75 neurotrophin receptor
- leucocyte immunoglobulin receptor B2 (LILRB2)
- triggering receptor expressed on myeloid cells 2 (TREM2)
- toll-like receptor 2 (TLR2R)
- neuroligin

A large group of proteins indirectly participate in the toxic effect of AβO:

- NMDA receptors
- AMPA receptors
- metabotropic Glu receptor 5
- frizzled receptor [112]
- alpha7nACh receptor
- adrenergic receptors
- calcium channels
- N-formyl peptide receptor 2
- IgGFc region receptor II b
- Transient receptor potential melastatin (TRPM2)
- insulin receptor
- Wnt receptor

AβOs also bind to synaptic proteins, such as synGap and Shank3 in the postsynaptic density (PSD), and these interactions may disturb the ordered structure of PSD [113].

It is not clear why there are so many receptor candidates for AβOs. It is suggested that not all of the above-mentioned proteins are genuine receptors for Aβ. AβOs can associate with many proteins owing to their IDP structure. Indeed, very recently, a systematic study of 15 AβO receptor candidates revealed that only three of them (PrPc, LilrB1, and neuroligin receptor 1) show specific binding to AβO [114]. PrPc and NgR1 preferentially bind synaptotoxic Aβ species, also in the brain of AD patients.

However, the main question of AβO–receptor interactions remains unanswered: how do these interactions cause cellular damage, and what is the mechanism of signal transduction? These mechanisms have been successfully analyzed in several cases.

**Figure 4:**
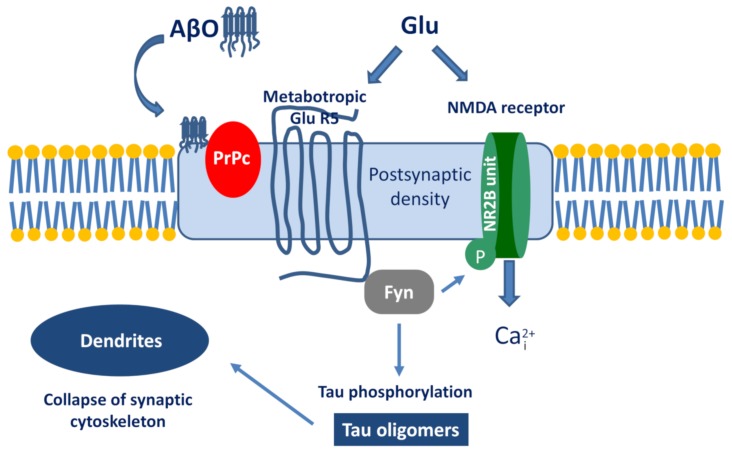
The signalization cascade initiated by the interaction between Aβ oligomer (AβO) and prion protein (PrPc).

The best known example is the PrPc–AβO interaction and the subsequent signalization cascade [115,116,117]. The simplified pathway is the following (Figure 4): AβO binds to PrPc, then stimulates the Proto-oncogene tyrosine-protein kinase Fyn by activating the metabotropic GluR5. Within the cell, Fyn can phosphorylate tau protein and also triggers the phosphorylation of the NR2B subunit of NMDA receptors, which activates Ca-influx. Taken together, the activation of the AβO-PrPc pathway has the following downstream consequences: Ca^2+^ dyshomeostasis, tau hyperphosphorylation, and synaptic dysfunction and loss.

A detailed analysis was also performed on how the NKAa3-AβO binding may cause neuronal damage [118]. It was demonstrated that signal transduction starts with a stepwise decrease of ATPase activity. AβOs initiate the restructuring of NKAa3 and formation of toxic membrane clusters with participation of different other proteins in the dendritic spines. Finally, Ca dyshomeostasis in the cell may cause mitochondrial dysfunction and apoptosis [119]. Very recent results support the hypothesis that inhibition of NKAa3 by AβOs starts the early stages of AD [113].

Recent experiments demonstrated that neuroligin R1 may also be a specific receptor for AβO, although the binding affinity is low [114]. Very recent studies shed light on the possible role of adrenergic receptors: AβO can bind to an allosteric site of the alpha2A receptor. This binding changes norepinephrine signaling and activates glycogen synthase kinase 3β (GSK3β), thereby triggering tau hyperphosphorylation [120].

Recently, the role of AβO in alterations of Glu-ergic signaling and cognitive decline during the progress of AD has been reviewed [121]. At the early stage of AD, AβOs upregulate tripatrite Glu-ergic synapses and thus increase Glu signaling forming excitotoxic environment around the neurons. The hypoactivation and cognitive decline in the later stage of AD are probably due to increasing neural loss.

It has been found very recently that the toll-like receptor 2 behaves as a primary receptor for AβOs and triggers the process of neuroinflammation [122]. Similarly, it was demonstrated that TREM2 (the triggering receptor expressed on myeloid cells 2) directly binds AβOs in nanomolar affinity and modulates microglial formation [123]. Certain nuclear receptors, such as the vitamin D receptors, are also linked to AβO toxicity and AD pathology [124].

In conclusion, several receptors might be involved in the mediation of AβO cytotoxicity in AD. It is proposed that the signalizations started by PrPc, NKAa3, TREM2, the glutamate, and ephrin receptors may play a decisive role in starting the pathophysiological processes.

### 2.5. Intracellular Aβ and Extracellular Plaques

It was found long ago that Aβ can form intracytosolic and intranuclear aggregates [125,126]. Early intracellular accumulation of Aβ (iAβ) was considered a key event of neurodegeneration, which predicts synaptic dysfunction [127].

It has been a controversial scientific debate on the origin and role of iAβ. There are alternate sources for iAβ formation. iAβ immunoreactivity was observed in neurons by light and fluorescence microscopy and immuno-EM during the life span of Down syndrome patients [128]. It can be formed within the cells. In addition, a part of iAβ may originate from extracellular sources entering the cells by receptor mediated internalization [127]. According to a very recent hypothesis, fast aggregation of long Aβs (43 to 52 AA residues) is the main component of the iAβ assemblies [129]. Subcellularly, the endoplasmic reticulum-intermediate compartment and the endosomal-lysosomal system participate in iAβ generation [130,131].

More and more evidence demonstrates that iAβ is responsible for early synaptic dysfunction and neuronal loss, plaque formation and cognitive impairment; in other words, iAβ is the toxic Aβ species [128]. It was demonstrated by immunochemistry that iAβ is able to form NFTs [132]. Accumulation of iAβ precedes tau hyperphosphorylation and is associated with microtubular degeneration. Mitochondria isolated from the brains of AD patients or AD mouse model animals contained iAβ. It is localized in the inner mitochondrial membranes and cristae [127]. Immuno-EM experiments explained the role of iAβ in plaque formation: plaques originate from degenerating neurites [128]. Activated microglia also plays an important role in the formation of amyloid plaques [128]. Very recent experimental data have also revealed that aggregated iAβ co-localized mostly with mitochondria and endosomes and much less with lysosomes in a 3xTg mouse model of AD [129]. Overexpression of Aβ and iAβ formation causes different mitochondrial dysfunctions (inner transport, axonal transport of mitochondria, and depletion in the synapses) [133]. iAβs may serve as a template showing intracellularly a prion-like seeding in N2A cell cultures [134].

The role of extracellular Aβ plaques in AD pathophysiology has been discussed for a long time. Plaques have been the main target of AD drugs, but plaque-decreasing therapies have failed. Recently, plaques are increasingly viewed as tombstones [128]. Immuno-EM experiments explained the role of iAβ in plaque formation: plaques originate from degenerating neurites [128]. Activated microglia plays also important role in formation of amyloid plaques [135]. Very recently, the nanoscale structure of plaques has been thoroughly studied [136]. The combination of two different 3D superresolution methods (array tomography (AT), and stimulated emission depletion microscopy (STED)) have been used for studying plaques in human AD brain tissues. It was observed that human amyloid plaques had a dense core of highly ordered Aβ structure (fAβ) surrounded by a peripheral halo of medium and small size of Aβ species. The latter diffusible, toxic AβO components of the halo may be responsible for the observed axonal degeneration in the neighborhood of the plaques.

Aβ also deposits intraneuronally as neurofibrillary tangles and also in blood vessels [137]. The structure and composition of amyloid deposits in the wall of blood vessels of CAA patients were studied by mass spectrometry very recently [138]. It was found that the molecular composition of Aβ deposits in blood vessels (mostly 1-40 and 2-40) are different to parenchymal deposits of AD patients (typically Aβ 1-42, Aβ 2-42, pGlu Aβ 3-42, pGlu Aβ 11-42).

## 3. Tau Proteins

TauOs and tau filaments may play a decisive role in the pathogenesis of AD and other tauopathies. Experimental studies have revealed the decisive role of TauOs in the pathogenesis of AD in the last ten years.

### 3.1. Tau Isoforms, Domain Structure, Post-Translational Modifications

The role of tau proteins in neurons was re-evaluated and excellently reviewed very recently [138,139,140]. Tau belongs to the family of microtubule-associated proteins (MAPs) that occur in brain cells. Alternative splicing of the human microtubule-associated protein gene (MAPT) product provides six tau isoforms ranging from 352 to 451 AA (Table 2).

There are two subfamilies of tau proteins: the three-repeat (3R) and the four-repeat (4R) groups, which possess three or four repeat domains. (Each repeat domain contains 18-amino acid sequences and is bound together by inter-repeat regions.) Tau proteins have important biological functions; their microtubule stabilizing role has been studied for a long time. A correct tau isoform ratio is necessary for maintaining brain cell homeostasis and preventing neurodegenerative diseases [141]. The repeat regions (together with short joining sequences) are the microtubule binding domain of tau. N-terminal truncation occurs at early stage of AD and provides a toxic 20–22 kDa tau fragment [142].

**Figure 5:**
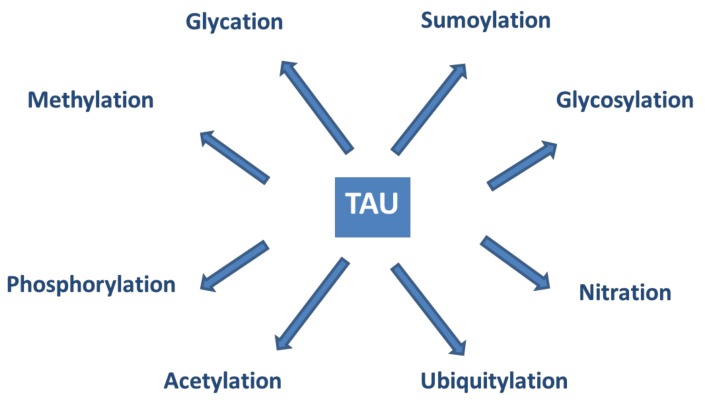
Post-translational modifications of tau proteins.

Tau proteins show a large number of post-translational modifications (PTMs) [142] (see Figure 5). PTMs may occur under both physiological and pathophysiological conditions. Phosphorylation occurs on the potential phosphorylation sites: the hydroxyl groups of serine (45 residues), threonine (35 residues), and tyrosine (5 residues). Acetylation, ubiquitylation, and sumoylation are enzymatic processes targeting one of the lysine residues of the 40 in tau and regulate tau activities and degradation. O-glycosylation occurs on Ser and Thr residues. Mostly, N-acetyl glucosamine is attached to tau. Only hyperphosphorylated tau can be N-glycosylated. Nitration of Tyr residues to 3-nitro tyrosine is mediated by reactive nitrogen species. Methylation of Arg and Lys residues is catalyzed by the enzyme methyl transferase and regulates tau metabolism competing with ubiquitylation. Glycation requires no enzyme catalysis. Carbohydrates are coupled covalently to tau in a pathological process leading to the formation of advanced glycation products, causing pathological consequences and neuronal death. It is assumed that PTMs mediate the structural diversity of different tauopathy strains [143].

Tau phosphorylation has been extensively studied as it changes the biological actions of the protein. Tau binds microtubules (MT) via special binding sites (3R and 4R domains). Up to now, at least 31 physiological phosphorylation sites have been identified. Phosphorylation of other sites (e.g., Ser 235, Ser 262, Ser 293, Ser 324, Ser 356, and Thr 231) may result in pathological hyperphosphorylated products. (The term hyperphosphorylation was introduced to distinguish between the normal and pathophysiological phosphorylations). It has been widely accepted that hyperphosphorylation decreases the binding of tau to MTs [144,145,146]. Several protein kinases can phosphorylate tau (e.g., GSK3β, DYRK1A, Fyn), and the phosphate groups can be cleaved by phosphatases (PP1, PP2A, PP2B). The fine balance between the kinase and phosphatase activities determines the phosphorylation state and biological activity of tau proteins.

Recent studies on familial AD patients have shown that the site-specific phosphorylation state of tau changes in different periods of disease progression [147]. Some sites are phosphorylated (e.g., formation of pTau217 and pTau181) as early as two decades before the development of aggregated tau-pathology. Others (e.g., pTau205) increase in parallel with brain atrophy and hypometabolism closer to the onset of AD symptoms. Further studies of the characteristic tau signature may facilitate the evaluation of the results of tau-targeted clinical trials.

### 3.2. Physiological Actions of Tau

Tau is a multifunctional protein that plays a role in basic physiological processes. Unexpectedly, tau deletion in mice does not cause big changes in the phenotype of the animals [148], although aged tau knock-out mice show synaptic loss and brain atrophy [149,150]. It is likely that young tau knock-out mice were protected against excitotoxicity by the accumulation of Syn-GAP1 [151,152].

The complex role of tau in health and disease has been reviewed by Naseri et al. [153]. Tau proteins have the following physiological functions:

*Protection of DNA*. Maintaining the integrity of the genetic material of neurons is highly important, owing to the longevity of this type of cells. Heat shock and oxidative stress cause tau-dephosphorylation and translocation into the nucleus [154]. Heat shock increases the tau-DNA interaction. Tau proteins bind mainly to the AT-rich regions of DNA.

*Modulation of NMDAR signaling.* Tau is involved in neurotransmission: It binds to Src-kinases, such as Fyn within the neurons [155]. Tau–Fyn interaction is required for NMDAR activation: Fyn- catalyzed phosphorylation of the NR2B subunits starts signaling [156]. Tau modulates signal transduction via modulating the function of presynaptic mitochondria that influence intracellular ATP and Ca-level [157].

The role of tau in *MT stabilization and spatial organization* has been thoroughly studied [158,159,160]. Tau plays a pivotal role in MT stabilization: it binds transiently to the C-terminal end of tubulin and drives polymerization of the protein to MTs [140,161,162]. Tau phosphorylation regulates tubulin binding to MTs [163,164]. According to a new hypothesis, tau is not an MT stabilizer, rather it increases the accessibility of long, labile domains for other stabilizer molecules [165].

*Phosphorylation of specific binding sites* (see Section 3.1) leads to dissociation of tau from MTs and oligomerization to toxic assemblies [166]. The precise mechanisms of tau-MT interactions (association and dissociation) have been intensively studied. The newest cryo-EM results provided a good model for the tau-tubulin binding [167].

*Axonal transport regulation.* In vitro studies demonstrate that tau modulates the motility of motor proteins in a concentration- and isoform-dependent manner [168]. Tau overexpression decreases the axonal transport of subcellular organelles, such as mitochondria [169].

*Interaction with the cytoskeleton*. Tau directly interacts with spectrin, an important cytoskeletal protein and thus regulates the shape of cells [170,171]. Tau interaction with the actin cytoskeleton results in the formation of actin filaments and bundles [172].

*Tau as a signaling molecule*. Regulation of the brain insulin signalization pathway is a novel role of tau [173,174]. Tau may control hippocampal plasticity via this pathway.

Native tau is involved in *normal synaptic activity* in the PSD [175]. In vivo observations demonstrate that native tau plays a role in *dendritic development* [139].

### 3.3. The Structure of Tau Oligomers and Fibrils

Tau in monomeric form is a highly soluble, intrinsically disordered protein with an open structure. A maximum of 10% of the protein sequence possesses any kind of ordered structure [176]. The native tau has only a low tendency for aggregation [177]. Free sulfhydryl groups in tau increase the predisposition for polymerization of the monomer. This is a multistep chemical process that shows different stages as reviewed in [141] (Table 3). According to the newest hypothesis, diffusible TauOs are toxic, but larger polymers probably are non-toxic assemblies [174]. 

TauOs have an increasingly ordered structure (β-sheet). The exact mechanism of TauO formation is not yet known [178]. Model peptide experiments emphasize the role of membranes in oligomerization [179]. There is an assumption that granular tau oligomers (gTauO) might be the most toxic species, this type of tau could be isolated from AD brain [139]. It is important that gTauOs are present in the brain at very early stages of AD, prior any cognitive symptoms [180]. It was hypothesized that Aβ assemblies catalyze tau aggregation by a cross-seeding mechanism [181].

**Figure 6:**
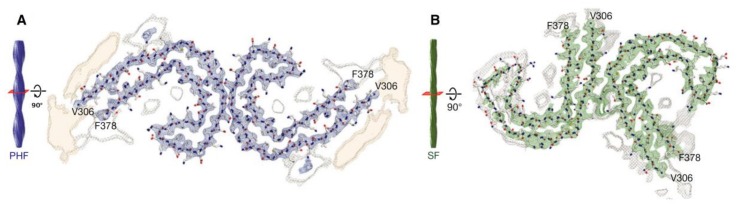
Cross-sections of paired helical and straight tau filament core structures from Alzheimer’s disease. Cryo-EM density and atomic models of paired helical (A) and straight (B) tau filaments. Sharpened, high-resolution maps are shown in blue (paired helical filaments) and green (straight filaments). Paired helical and straight filaments are ultrastructural polymorphs. Their protofilament corresponds to residues V306-F378 of tau (R3 + R4 + 10 amino acids carboxy-terminal to the repeats). Unsharpened densities are shown in orange and grey. From [185]. Reprinted with permission from the publisher (John Wiley & Sons, Inc.).

Tau fibrils form intracellular deposits (e.g., NFTs). The determination of the structure of tau fibrils (straight filaments and PHFs) at atomic level by cryo-EM represents a real breakthrough in the field. Fitzpatrick isolated tau amyloid fibrils (both straight and paired helical filaments) from an AD brain and analyzed the conformation by cryo-EM (Figure 6). At 3.4 Å resolution, tau in NFTs is aberrantly misfolded and hyperphosphorylated [182]. The ultrastructural level of the two filaments were a little different but had a common element: C-shaped monomers formed β-sheet conformation, and the cores of both fibrils contain this β-sheet motif. Filaments are formed primarily from R3 and R4 repeat regions of tau. The filaments from Pick’s disease brain [183] contained only three R tau domains. Filaments were also isolated from chronic traumatic brain encephalopathy brains [184], and the structure was determined at 2.3 Å resolution. 3R and 4R domains form the ordered core structures that contain a hydrophobic cavity in these tau filaments. These results show that tau have different phosphorylation stages and form distinct conformers in different neurodegenerative diseases. Very recently, it was demonstrated that PTMs mediate the structural diversity of distinct tauopathy strains [143].

### 3.4. Pathophysiological Effects of Tau Assemblies.

The view on the role of different tau assemblies in AD pathogenesis has changed very similarly to the stepwise changing of the Aβ hypothesis. By now, it has been widely accepted that the big tau assemblies (straight filaments, PHF, NFT, and ghost tangles) are the less toxic tau forms, and rather the smaller, diffusible oligomers are involved in AD pathogenesis [174]. Synaptic dysfunction and loss, as well as axonal transport disturbances, are linked to TauOs and not to NFTs, although tau fibrils may physically hinder transport processes within the cells. The level of cognitive decline and synaptic and neuronal loss in AD is most closely correlated with tau pathology. PET imaging using flortaucipir labeling of brain-tau in AD patients demonstrated that tau is concentrated precisely where brain atrophy is most severe [186].

Several pathophysiological actions of TauO have been observed: 

*Disaggregation of microtubules.* Different pathological conditions (e.g., AβO accumulation, GSK3β activation) cause tau modifications (e.g., hyperphosphorylation) and formation of soluble oligomers. As a result, tau dissociates from the MTs (Section 3.2). The structure of MT collapses, and tau may simultaneously translocate to synapses. The details of tau–microtubule interactions, the dynamism of adsorption–desorption (detachment) processes, has been analyzed very recently [160]. Big tau aggregates may be involved in axonal transport defects, causing a direct physical blockade of the transport [187].

*Loss of DNA protection.* TauOs are not able to enter the cell nucleus, and thus, they cannot protect nuclear DNA [188].

Pathological tau species may impair the transport of nucleocytoplasma [189].

*Increased excitability of neurons*. Toxic TauOs modulate the neuronal activity and increase their excitability. AD is characterized by epileptic seizures [190]. Administration of tau antisense RNA to AD mice reduce TauO formation and decrease the high mortality of the animals [191,192].

*Synaptic loss and disturbing neural circuits.* Stable TauO aggregates may accumulate in huge amount in cortical synapses (observed in brightfield microscopy) [193]. Dendritic tau disrupts the synaptic cytoskeleton [194] and causes synaptic dysfunction and loss. TauO suppressed and silenced many neurons and impaired the integrity of neural circuits in APPxPS1 mice [195].

*Neuroinflammation*. Studies on human brains demonstrated that Tau can be involved in microglia activation and, thus, in neuroinflammation [196]. Tau pathology is associated with chronic neuroinflammation, and microglia may phagocyte the pathological dysfunctioning synapses in tauopathic brain [197].

*Cell-to-cell spreading*. Synaptic tau is a transmissible pathogen: TauOs may disseminate the misfolded tau after templated propagation by cell-to-cell spreading along the anatomical connections. It was observed recently that tau propagation may occur via exosomes [198]. Microvesicles or ectosomes might be also involved in tau spreading [199]. The mechanisms of the propagation of tau in cellular and animal models of diseases have been recently reviewed [200]. No possible receptors have been found that facilitate tau internalization, rather it occurs via macropinocytosis.

According to several authors, AD might be an infectious disease within the living brain [201]. Very recent observations support the cell-to-cell spreading of pathological tau, however, exclude the behavior of TauOs as infectious agents in cell cultures and animal models [202]. Clinical studies demonstrated that tau pathology spreads hierarchically throughout the cortex, and higher network connectivity increases tau propagation [203].

## 4. Genetic Risk Factors of AD: Classical Studies and Novel Results

The genetic risk of AD should be reconsidered after understanding the physiological and pathophysiological role of AβO and TauO. The genetic background of familial, autosomal dominant AD (ADAD, a subset of EOAD) is well established. Overproduction of Aβ peptides and its decreased clearance augment Aβ level in the brain, and Aβ-aggregation and formation of toxic AβO occur over a critical concentration. ADAD is linked to mutations within the APP and PS1 genes [204]. The mechanisms by which these mutations are coupled to AD have been reviewed [18]. The effects of pathogenic and non-pathogenic mutations of the Aβ-sequence within the APP have been studied, and eight important mutation sites were found [25]. Several mutations (e.g., A02V) induce Aβ overproduction by modulating APP processing, causing amyloid formation and early onset dementia [205]. Interestingly, the so-called Icelander mutation in the same site of the sequence reduces APP cleavage to Aβ, and thus, A02T proved to be a non-pathogenic, protective mutation [206].

Several mutations modulate the conformational stability, and thus, the toxicity of Aβ. The English APP mutation (H06R) is pathogenic as the peptide may operate as a seed for oligomerization and formation of toxic β-structure [207].The Flemish (A21G), Dutch (E22Q), Italian (E22K), and Iowa (D23N) mutations occur in the central hydrophobic core region of Aβ and affect the conformation stability of Aβ by modulating salt-bridge formation between Asp22 and Lys28 [25].

The Flemish, Dutch, and Italian mutations cause presenile dementia and cerebral hemorrhage. The Iowa and Piedmont (L34V) mutations are associated with dementia and CAA [208]. Until now, a lot of coding mutations have been found in the APP gene. Most of them are pathogenic and cause ADAD. However, these autosomal dominant hereditary disease variants represent only 0.5% of AD cases and cannot explain the genetic background of LOAD.

The genetic background of LOAD is much more complicated. Although epidemic studies show that about 65% of a person’s risk for AD is genetically determined, until now only a few dozen AD risk genes have been identified. Theoretical considerations reveal that there could be hundreds of additional genetic variants, each of which contributing in a small but significant way to AD risk.

Apolipoprotein E (ApoE) is the strongest risk factor for LOAD [209]. This protein binds lipids and transports them to target sites. The APOE gene has three major alleles (ε2, ε3, and ε4), the proteins differ from each other by one or two amino acids. The allele frequencies are different: ~7, 79 and 14% for ε2, ε3, and ε4, respectively. The APOE status is predictive for late onset AD [210], approximately 40% to 65% of LOAD patients have at least one APOE ε4 allele. ApoE proteins regulate AD risk in different pathways [16]:

a) Aβ-dependent pathways (reviewed in [210]). ApoE proteins interact with Aβ by an isoform-specific efficacy (ApoE2 > ApoE3 > ApoE4). ApoE4 supports Aβ aggregation and deposition [211]. ApoE indirectly regulates Aβ clearance by competitive binding to Aβ-receptors, e.g., LRP1.
b) ApoE also modulates tau pathology; especially ApoE4 enhances tau pathogenicity.
c) ApoE4 mediates AD risk by modulation of immune and microglial responses to amyloid plaques.
d) ApoE4 has direct pathological effects on neurons and neuronal networks. ApoE3 expressions in neurons are protective by stimulating neurite outgrowth.
e) ApoE4 directly impairs the blood-brain-barrier function in AD.

In recent years, genome-wide association studies (GWASs) reinforce that AD is a complex disease, in which APP processing and immune response play key roles [212]. Whole genome sequencing (WGS), GWAS, and gene-expression network analysis proved that LOAD genetics implicates microglial pathways and CR1, SII1, MS4As, TREM2, ABCA7, CD33, INPP5D genes are associated with disease risk [212]. The results of the largest meta-GWAS approach (analyzing 19,089 AD cases) and follow-up analysis for AD risk has been published very recently [212]. It was found that altogether 39 genetic variants may be associated with AD risk (beyond the APOE variants). Polygenic risk scores (PRS) were used for identification of the following novel genetic variants: APP (new variants), SHARPIN, PRKD3/NDUFAF7, CHRNE, PLCG2 locus mutations.

The PRS approach could be a robust tool to predict the risk and the age of onset of AD. The latest studies on two AD mouse models demonstrate that microglia play a key role in AD pathogenesis, and AD risk genes converge to microglia [213]. If microglia were exposed to Aβ, novel genes have been significantly upregulated and expressed and, as a result, the microglia switched to an activated status. This unique gene expressing module included 11 novel genes (GPC2, TREML2, SYK, GRN, SLC2A5, SAMSN1, PYDC1, HEXB, RRBP1, LYN, and BLNK). It was hypothesized that the transition of AD from the prodromal, biochemical to clinical phase is determined by the combined inheritance of low-penetrant SNPs (Single Nucleotide Polymorphism), and multiple SNPs within the functional network push the homeostatic balance to disease-causing disturbances. The classical gene-based view of AD etiology should be changed.

## 5. Aβ-Tau Crosstalk

Several important questions are still to be answered in the Aβ-tau relationship. It has not been fully understood how toxic actions of AβO and TauO proceed. First, it was supposed that only Aβ triggers and regulates tau pathology in AD. However, there are many tauopathic diseases without any Aβ occurrence. Recent data show that cholesterol metabolism, endosomal trafficking, and ApoE and microglial activation can regulate tau pathology [214] in AD, independently from Aβ.

Aβ-tau crosstalk plays an important role in iron homeostasis of the brain [140]. The presence of soluble tau is necessary for promoting APP trafficking to the neuronal surface; this process lowers iron levels and prevents iron accumulation in the brain.

It is assumed that AβO formation precedes the hyperphosphorylation of tau [215,216], and Aβ pathology increases the production of tau [217]. It is also widely accepted that the two proteins have synergistic effects on AD progression. Many studies suggest that biological actions induce both pathologies via correlated, but independent pathways. AβO may activate GSK3β that plays a key role in the hyperphosphorylation of tau. This modification changes the tau-tubulin binding, resulting in detachment of tau from the MT and TauO formation [218].

Several research groups studied tau-Aβ cross-seeding [219,220] and found that aggregated Aβ promoted the formation of tau aggregates. It was demonstrated that AβO enhances tau seeding in mouse neurons [221,222] and facilitates intracellular tau aggregation by promoting the uptake of tau seeds. However, transduced Aβ fibrils slightly reduce tau seeding.

It has been found very recently that tau and Aβ cooperate in a mouse model, causing hyperactivity and transcriptional perturbations [223]. Both Aβ and tau affects the activity of neuronal circuits. Aβ promotes neuronal hyperactivity, while tau suppresses it [197]. Tau silenced most of the neurons and suppressed Aβ-dependent neuronal hyperactivity in an AD mouse model (APPxPS1 mice). Neuronal silencing dominated over Aβ-caused hyperactivity; however, only the soluble TauO had sufficient effect. The NFTs were inactive.

Current studies show that neuroinflammatory pathways also link together Aβ and tau effects. In microglia, if cells contact with Aβ, the inflammasomes become active and induce inflammation. However, NLRP3 inflammasome activation drives tau pathology by increasing tau accumulation within the neurons [224]. A visible link exists between Aβ and tau: inflammatory processes promote Aβ-pathology and then also tau-pathology [198].

## 6. Conclusions and Future Directions

We do not have satisfactory knowledge on the mechanisms underlying the disease—this is the biggest problem in AD drug research. Two facts make Alzheimer’s drug design an unbelievably complicated task: 1) Both Aβ and tau proteins have pivotal physiological functions in the brain cells. 2) A small change in protein homeostasis (formation of ordered conformations, post-translational modifications) brings about formation of invisible toxic oligomers. Microscopically visible Aβ and tau deposits are no genuine AD drug targets, and thus, the application of the original amyloid cascade hypothesis for drug design has limitations. In the last couple of years, a high number of AD drug development trials aiming to eliminate Aβ plaques has failed [225,226,227,228]. (Only aducanumab, an AβO-specific antibody raised some optimism in clinical trials.) These failures may be originated from the fact that monomeric Aβ plays important physiological functions, and some therapies might decrease Aβ to a very low level. It is also possible that drug treatments begun too late, and the neurodegenerative processes were already irreversible. It has probably been a mistake to target amyloid plaques that now are considered to contain mainly non-toxic Aβ fibrils and are also present in the brain of healthy individuals. It is possible that Aβ accumulation is only a reaction of brain cells to their damage [24].

Drug research targeting tau assemblies received less attention. Nowadays, AD therapy is moving from Aβ to tau. By now, novel physiological tau functions have been discovered that provide novel targets.

Biological aging bringing about protein dyshomeostasis is the most important risk factor of AD. One should understand why the aging brain is so vulnerable to AD pathogenesis. The results of biological gerontology provide targets for slowing down the aging process and prevent the early beginning of AD [229].

Novel developments in AD biomarkers and imaging methods provide excellent tools to follow and monitor the effectivity of new drug candidates in clinical trials. Tau-PET imaging with Flortaucipir may demonstrate and predict location of tau assemblies in AD patients [186]. New AD animal models represent better simulation of the human disease.

Nowadays, the following targets and directions seem to be the most important ones for novel AD drug developments:

*IntracellularAβ oligomers (iAβOs).* Neutralizing antibodies, specific for AβO, ought to bind both extra- and (after entering the cells) iAβOs. Small peptidomimetic molecules that disaggregate oligomers to non-toxic monomers could also be used [230]. Maintaining the partly helical structure of Aβ monomers by helix stabilizing agents is under development. 

Neutralizing *intracellular TauOs* might be the most promising target for AD drug research. Monoclonal ABs specific for TauO should be developed; however, these big molecules ought to enter the neurons. This process requires also the development of suitable vector molecules.

Dysfunctional resolution of chronic neuroinflammation is now considered as a driver of AD pathogenesis. Resolution of neuroinflammation by pro-homeostatic lipids (called specialized pro-resolving mediators, SPMs) might be a rational treatment in early stages of AD [231].

Restoration of protein homeostasis may be also rational drug targets. Tackling mitochondrial, metabolic, and vascular dysfunctions, modulating autophagy, preventing synaptic dysfunction, and increasing the clearance of amyloid proteins represent the therapies that slow down the aging process.

Aβ and tau interdependence is important in AD development and progress. In the future, a combination therapy, namely simultaneous administration of tau-targeting and Aβ-targeting drugs may be a useful remedy for AD treatment.

A rapid and successful development of cell-replacement therapies (e.g., application of multipotent stem cells) is expected for AD treatment [232].

## Figures and Tables

**Table 1:** Characterization of the two subpopulations of Aβ.

*Toxic Subpopulation*	*Non-Toxic Subpopulation*
Mw > 50 kDa (HMW, high mol. weight)	Mw < 50kDa (LMW, low mol. weight)
unrelated to plaques	related to plaques
no reaction with anti fAβ antibody	binding anti-fAβ antibody
“type 1” AβO: toxic	“type 2” AβO: non-toxic
disrupt memory function	no effect on memory function

AβO: Aβ oligomer, fAβ: fibrillar β-amyloid.

**Table 2:** Isoforms of the tau protein.

Name	MW (kDa)	Length (AA)			
h tau 40	45.9	441	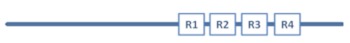	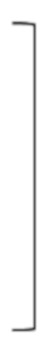	
h tau 34	43.0	412	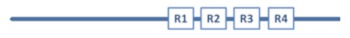		**4R**
h tau 24	40.0	383	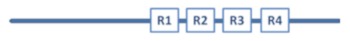		
h tau 39	42.6	410	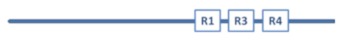	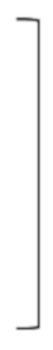	
h tau 37	39.7	381	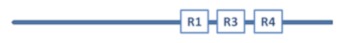		**3R**
h tau 23	36.7	352	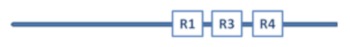		

**Table 3:** Classification and toxicity of tau oligomers and fibrils [174].

Name	Size	Toxicity
Tau monomers	67–70 kDa (352–441 AA)	non-toxic
Abnormally phosphorylated monomer	67–70 kDa	toxic
Tau dimer-trimer	120–180 kDa	toxic
Small soluble oligomers (TauOs, 6–8 units)	300–500 kDa	toxic
Granular tau oligomers (gTauOs, 36 units)	1800 kDa	toxic
Straight filaments (SFs)	50 nm x 10 nm	not always toxic
Paired helical filaments (PHFs)	80 nm x 10–20nm	probably non-toxic
Neurofibrillary tangles (NFTs)	“	probably non-toxic
Ghost tangle (originated from degenerated neurons)		probably non-toxic

## Abbreviations

Aβ oligomer (AβO), Alzheimer’s disease (AD), amyloid β protein precursor protein (APP), antibody (AB), β-amyloid (Aβ), electron microscopy (EM), fibrillar β-amyloid (fAβ), glycogen synthase kinase 3 (GSK-3β), intracellular β-amyloid (iAβ), intrinsically disordered protein (IDP), microtubule (MT), prion protein (PrP), tau oligomer (TauO).
